# Tumor-Stroma Ratio in Basaloid and Conventional Laryngeal Squamous Cell Carcinoma: Prognostic Significance and Concordance in Paired Biopsies and Surgical Samples

**DOI:** 10.3390/cancers15061645

**Published:** 2023-03-07

**Authors:** Gino Marioni, Stefano Taboni, Marta Sbaraglia, Leonardo Franz, Tommaso Saccardo, Anna Colombo, Camilla Zimello, Anna Chiara Frigo, Marco Ferrari, Lara Alessandrini

**Affiliations:** 1 Otolaryngology Section, Department of Neuroscience DNS, University of Padova, 35100 Padova, Italy; 2 Guided Therapeutics (GTx) Program International Scholarship, University Health Network (UHN), Toronto, ON M5G1L7, Canada; 3 Artificial Intelligence in Medicine and Innovation in Clinical Research and Methodology, Department of Clinical and Experimental Sciences, University of Brescia, 25100 Brescia, Italy; 4 Pathological Anatomy Unit, Department of Medicine DIMED, University of Padova, 35100 Padova, Italy; 5 Phoniatrics and Audiology Unit, Department of Neuroscience DNS, University of Padova, 31100 Treviso, Italy; 6 Department of Cardiac-Thoracic-Vascular Sciences and Public Health, University of Padova, 35100 Padova, Italy; 7 Technology for Health, Department of Information Engineering, University of Brescia, 25100 Brescia, Italy

**Keywords:** basaloid squamous cell carcinoma, larynx, tumor-stroma ratio, biopsies, surgical specimens, concordance, prognosis

## Abstract

**Simple Summary:**

This retrospective study is the first attempt to investigate the tumor–stroma ratio in a series of laryngeal basaloid squamous cell carcinomas (SCCs), comparing them with a group of stage-matched conventional SCCs, in both preoperative and surgical specimens. This study’s aim was to ascertain the biological aggressiveness of laryngeal basaloid SCCs and to investigate any possible role of stromal-related features in such a clinical behavior. The tumor–stroma ratio, evaluated in laryngeal biopsies and in the entire excised tumor, displayed a prognostic effect in terms of reduced disease-free survival in conventional SCC cases but not in basaloid ones.

**Abstract:**

Basaloid squamous cell carcinoma (BSCC) is a subtype of squamous cell carcinoma (SCC) associated with a poor prognosis. Tumor–stroma ratio (TSR) has been introduced as a prognostic feature in many solid tumors. TSR was investigated in a series of laryngeal BSCCs and compared with a group of stage-matched conventional SCCs (cSCCs), in both preoperative and surgical specimens, with the intent of ascertaining the more aggressive behavior of BSCC and verifying the presence of stromal-related causes. A series of 14 consecutive laryngeal BSCCs and a control group of 28 stage-matched conventional cSCCs were analyzed. A higher nodal metastasis presence was found in BSCCs (57.1% vs. 28.6%). The recurrence rate was 33.5% and 63.6% in the cSCC and BSCC groups; disease-free survival (DFS) was higher, though not significantly, in patients with cSCC. TSR, large cell nests, and tumor budding showed a moderate to very good agreement, and stroma type a good to very good agreement between biopsies and surgical specimens in the cSCC group. In the BSCC group, agreement was poor to very good for TSR and stroma type, and good to very good for large cell nests and tumor budding. Age was the only feature significant in predicting recurrence in the BSCC group (*p* = 0.0235). In cSCC, TSR low/stroma rich cases, when evaluated on biopsies or surgical specimens, were associated with lower DFS (*p* = 0.0036; *p* = 0.0041, respectively). Laryngeal BSCCs showed a lower DFS than cSCCs, even if statistical significance was not reached. TSR, evaluated in laryngeal biopsies and excised tumors, was prognostic in terms of DFS in cSCC but not in BSCC cases.

## 1. Introduction

Basaloid squamous cell carcinoma (BSCC) is a subtype of squamous cell carcinoma (SCC) with prominent basaloid morphology and the presence of squamous differentiation, often with myxoid or hyaline stromal alterations [1]. The term was first used in 1986 [2], and BSCC was included in the 1991 edition of the Histological Typing of Tumours of the Upper Respiratory Tract and Ear by the WHO [3]. Although BSCC was originally defined as an aggressive subtype, more contemporary analyses revealed similar recurrence and survival rates between BSCC and conventional SCC when patients are matched by primary head and neck site and stage [4]. Linton et al. [5] examined the retrospective data of a population-based registry from the Surveillance, Epidemiology, and End Results (SEER) database, including 34,196 patients treated between 2004 and 2009 with head and neck primary SCC (*n* = 33,554) and BSCC (*n* = 642). They concluded that the BSCC subtype was not an independent adverse prognostic factor. Using the SEER database, Fritsch and Lentsch [4] identified 1083 head and neck BSCC patients and 66,929 conventional SCC patients, diagnosed between 2000 and 2008. On multivariable analysis, disease-specific survival (DSS) was significantly better in the oropharyngeal BSCC group. Conversely, DSS was worse for laryngeal BSCC. DSS was similar among patients with sinonasal, nasopharyngeal, hypo-pharyngeal, and oral tumors. 

Tumor aggressiveness is significantly influenced by the tumor microenvironment, the main component of which is peritumoral stroma. Crosstalk between the neoplastic cells and the associated stroma contributes to tumor progression and metastasis [6]. Thus, tumor-related stroma could provide novel insights into the behavior of malignancies. The tumor–stroma ratio ([TSR], the proportion of tumor tissue relative to surrounding stromal tissue), has been described as a valuable prognostic feature in many solid tumors [7], with higher stromal content/lower TSR (that is, more than 50% of stroma, as proposed and standardized by international guidelines [8]) being associated with a worse prognosis. The simple, quick, and cost-effective assessment, the inter-observer reproducibility [8,9], and the good concordance between biopsies and matched surgical samples [10] supported the role of TSR as a valuable prognostic parameter also in head and neck malignancies [9,11] including laryngeal carcinoma [10,11,12,13,14]. Besides TSR, other more conventional histopathological features that are theoretically associated with the interplay between tumor and stroma, that is, the tumor growth pattern and the presence of a different stromal reaction, of neoplastic large nests, and of tumor buds, have demonstrated a promising prognostic value in conventional laryngeal SCC [10,11,12,13,14].

This retrospective study has been the first attempt to investigate TSR in a series of 14 consecutive laryngeal BSCCs and to compare findings with a control group of 28 stage-matched conventional SCCs, in both preoperative and surgical specimens, with the intent of ascertaining the more aggressive biological behavior of laryngeal BSCC and verifying the presence of stromal-related causes.

## 2. Materials and Methods

### 2.1. Patients

The study was conducted respecting the Helsinki Declaration. A detailed informed consent form was signed: patients agreed to “the use of their clinical data for scientific research purposes in the medical, biomedical and epidemiological fields, also in order to be recalled in the future for follow-up needs”. Data were examined in accordance with the Italian privacy and sensitive data laws.

The investigation involved 14 consecutive laryngeal BSCCs (diagnosed in a period ranging from 2000 to 2020) and a control group of 28 stage-matched conventional SCCs (diagnosed in the period 2000–2013) treated with curative intent. The vast majority of patients in the two groups were cigarette smokers. All patients underwent transoral endoscopic evaluation with biopsy, neck ultrasonography (US) (with or without fine needle aspiration cytology), morphological imaging (head and neck contrast-enhanced computerized tomography [ceCT] and/or magnetic resonance), chest X-ray, and liver US. In the most recent cases, thorax-abdomen ceCT was used instead of chest radiography and liver US.

All patients underwent laryngeal surgery at the Section of Otolaryngology of Padova University, including unilateral or bilateral cervical lymph node dissection in 13 BSCC cases and 27 conventional SCC cases. On final histopathological examination, the laryngeal surgical margins were negative in all cases. Pathological findings warranted postoperative adjuvant RT (with or without concomitant chemotherapy) in 7 BSCC cases and 12 conventional SCC cases. All patients who had received an indication for postoperative RT completed the treatment program. No distant metastases were detected at diagnosis. Clinical follow-up visits after treatment at our institution were scheduled as previously described [10]. ceCT of the neck, total body positron emission tomography, chest CT, and neck and liver US were repeated when clinically indicated. The mean follow-up was 59.7 (SD = 69.9) and 71.6 (SD = 49.9) months (median 22, range 9–230 months and 63.5, range = 10–192 months) in BSCC and conventional SCC cases, respectively.

### 2.2. Histopathological Investigations

Two dedicated head and neck pathologists (LA and MS) reviewed all available H&E stained slides from both groups of patients: slides from preoperative biopsies were available for 9 out 14 BSCC cases; in the other 5, the biopsy was performed in another institution. Biopsy material was available for all the conventional laryngeal SCCs considered as a control group. For surgical samples, the most appropriate slide for each specific histopathological variable considered was identified (see below). 

#### 2.2.1. TSR Assessment

Using a 5× objective, areas with the greatest amount of visible stroma were selected in preoperative biopsies and paired surgical samples. One area with both tumor and stromal tissues within this vision field was selected using a 10× magnification. The tumor cells had to be visible on all 4 sides of the selected image field, as required by van Pelt et al. [8]. The field section with the highest percentage of stroma was selected for the final estimation of the TSR: a single field with a stroma-rich score was decisive and sufficient to be included in the stroma-rich category. For statistical analysis, stromal ratio groups were divided into stroma-rich and stroma-poor groups. Stroma-rich tumors were defined as those with >50% of stroma, and stroma-poor/TSR high tumors were defined as those with ≤50% of stroma in the defined field. All areas with necrosis, major vascular structures, and muscle tissue were considered unsuitable for scoring, whereas nerves, smaller vascular structures, and lymphocytic infiltration were not excluded from the stromal compartment analysis. 

#### 2.2.2. Tumor Budding Assessment

Tumor budding was defined as single cells or clusters of up to four cells at the invasive margin of the cancer; it was evaluated on the surgical specimens’ slides as peritumoral budding (buds at the invasive front of the tumor), and on the biopsies’ slides as intratumoral budding (buds in the tumor core). The International Tumor Budding Consensus Conference (ITBCC) guidelines proposed three different budding scores, defined as Bd1, Bd2, and Bd3, consisting of 0–4, 5–9, and 10 or more buds in a hotspot of 0.785 mm^2^, respectively [15]. For statistical reasons, a cut-off of five was applied to distinguish between low risk (LR; fewer than 5 buds, equal to Bd1) and high risk (HR: 5 or more buds, equal to Bd2 and Bd3) cases, a criterion routinely applied in head and neck carcinoma [9]. 

#### 2.2.3. Other Histopathological Variables Evaluated

Tumor infiltrating lymphocytes (TILs) and tumoral growth patterns were assessed only on surgical samples, whereas large cell nests, defined as clusters of more than 15 tumor cells surrounded by stroma [13], were evaluated in both biopsies and surgical specimens, using a binary system (0 = absence; 1 = presence). TILs were reported as the percentage of total intratumoral/peritumoral stromal area occupied by mononuclear inflammatory cells (lymphocytes and plasma cell). Tumoral growth patterns were defined as follows:—expansive cases included tumors with well circumscribed borders without normal tissue within the tumor and/or with only a few large neoplastic nests at the invasive front;—the infiltrative pattern was characterized by poorly circumscribed borders and obvious stromal invasion by nests, cell clusters, or single cells spreading into normal tissue.

### 2.3. Statistical Analysis

SAS 9.4 for Windows (SAS Institute Inc., Cary, NC, USA) was preferred for the statistical analyses. The data are reported as mean and standard deviation (SD), median, and range for quantitative variables and as count and percentage for categorical variables.

BSCC and SCC groups were compared with the Student’s *t*-test or the Mann–Whitney test as appropriate in the case of quantitative variables and with the chi-square or Fisher’s exact test for categorical variables.

The concordance of TSR scoring on SCCs and BSCCs between biopsy and surgical specimens was evaluated with the Gwet’s AC1 statistic. It adjusts the overall agreement probability for chance agreement. The Gwet’s AC1 statistic has the same interpretation of the kappa statistic in the following range: <0.20, poor agreement; 0.21 to 0.40, fair agreement; 0.41 to 0.60, moderate agreement; 0.61 to 0.80, good agreement; 0.81 to 1.00, very good agreement.

The disease-free survival (DFS) was measured as the time from treatment completion to SCC or BSCC recurrence or to the last follow-up evaluation for censored patients. 

The prognostic role of each clinical-pathological feature on recurrence-free survival was calculated with univariate Cox regression. Firth correction was applied in the case of undefined maximum likelihood estimates.

The potential predictors resulting as statistically significant in the univariate analysis were considered in a multivariate Cox regression model. When both biopsies and histology resulted as statistically significant, since they are associated, biopsies were disregarded.

The plot of the cumulative Martingale residuals against the values of the covariate and Kolmogorov-type supremum test based on a sample of 1000 simulated residual patterns was used to test the proportionality for quantitative covariates. The results have been expressed as *p*-value and hazard-ratio (HR) with a 95% confidence interval (CI).

A *p*-value < 0.05 was considered indicative of statistical significance.

## 3. Results

### 3.1. Patients’ Demographic and Clinical Characteristics

The overall distribution of demographic and clinical features, as well as their differences between basaloid and conventional SCC groups, are summarized in Table 1. The age at diagnosis was significantly higher in the basaloid group compared to conventional SCCs (mean (SD): 70.9 (8.5) vs. 64.9 (8.6) years; *p* = 0.0405).

### 3.2. Concordance between Pathological Variables Measured on Biopsies and on Surgical Specimens in Conventional SCCs and BSCCs

**Figure 1:**
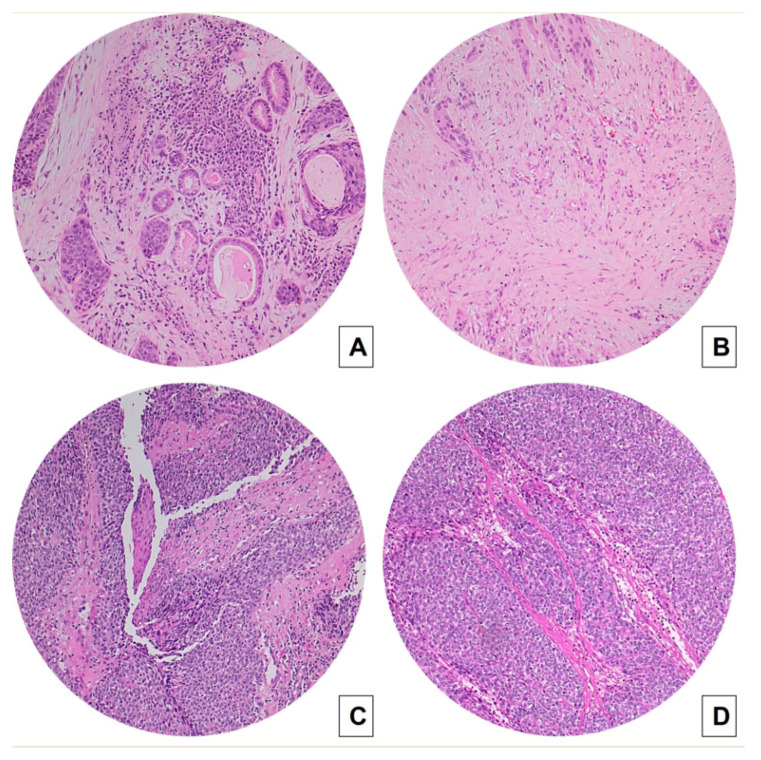
Concordance between TSR measured on biopsies (**A**,**C**) and on matched surgical specimens (B,D) in conventional SCC (**A**,**B**) and BSCCs (**C**,**D**) (hematoxylin and eosin H&E stained slides, original magnification 100×). Laryngeal BSCC (**C**,**D**) shows a high TSR (stroma-poor case), whereas conventional SCC (**A**,**B**) displays a low TSR (stroma-rich case).

**Figure 2:**
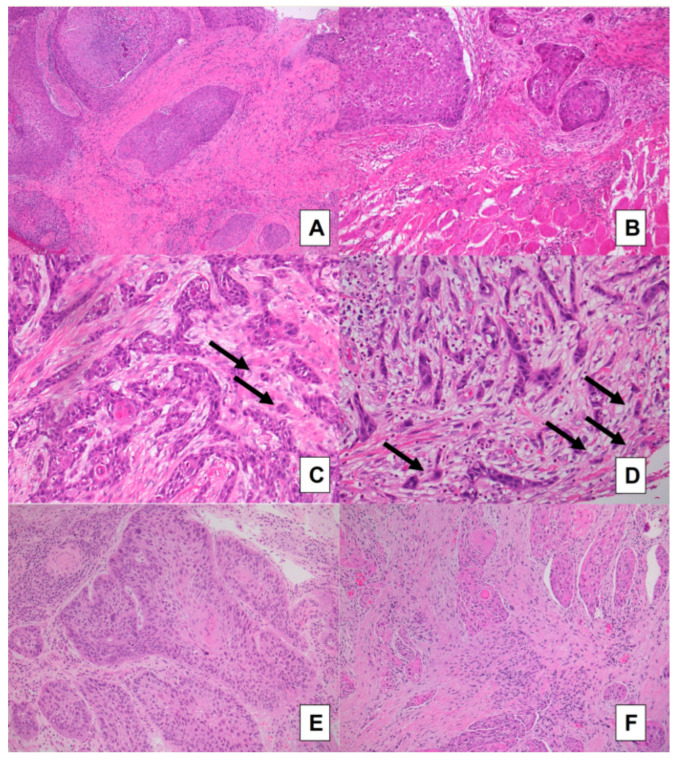
Conventional SCC cases: comparison between preoperative biopsies (**A**,**C**,**E**) and matched surgical samples (**B**,**D**,**F**). Concordance was good for the assessment of large neoplastic nests and very good for the evaluation of stroma type: a case with a more fibrotic stroma and large cell nests is evident in images (**A**,**B**), and a case with a more fibroblastic stroma is presented in images (**E**,**F**). Tumor buds are highlighted by black arrows in intratumoral stroma of a biopsy (**C**) and at the front of invasion in paired surgical specimen (**D**) (H&E stained slides; (**A**,**B**,**E**,**F**) original magnification 100×; (**C**,**D**) original magnification 200×).

In patients with conventional SCC, the agreement between biopsies and surgical specimens was moderate to very good for TSR (Figure 1A,B), large cell nests (Figure 2A,B), and tumor budding (Figure 2C,D) (AC1 statistic: 0.7466 [95% CI 0.5028–0.9904], 0.7225 [95% CI 0.4736–0.9714], and 0.7162 [95% CI 0.4750–0.9574], respectively), and it was good to very good for stroma type (AC1 statistic: 0.8629 [95% CI 0.7016–1.000]) (Figure 2A,B,E,F).

**Figure 3:**
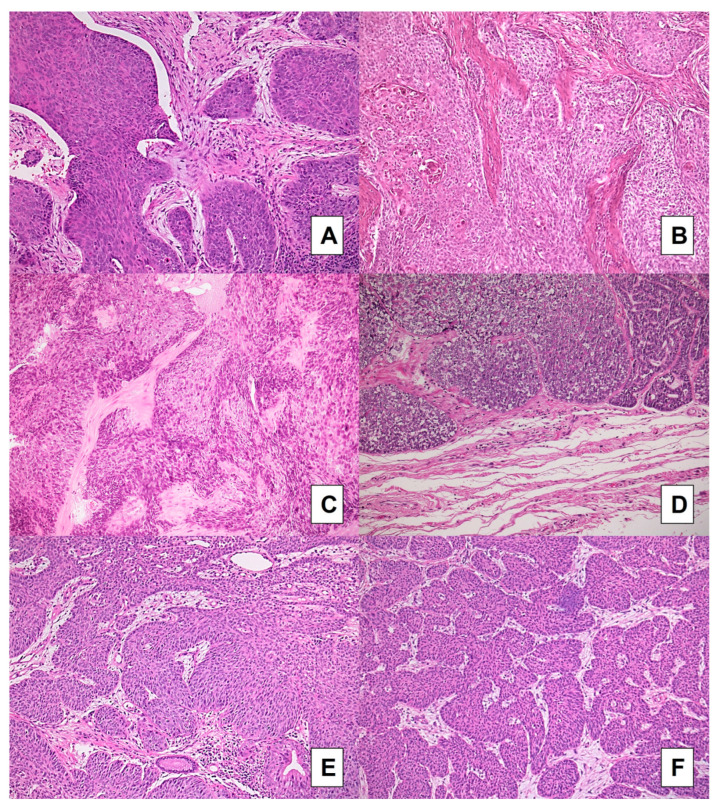
BSCC cases: comparison between preoperative biopsies (**A**,**C**,**E**) and matched surgical samples (**B**,**D**,**F**). Concordance was very good for the evaluation of tumor budding and for the assessment of large neoplastic nests: nearly all cases showed absence of budding and presence of large nests; a case (**A**,**B**) showing a fibroblastic stroma in preoperative biopsy (**A**) but a fibrotic stroma type was found in paired resection specimen (**B**); the same fibroblastic stroma was detected in another case in both biopsy (**E**) and matched resection specimen (**F**) (H&E stained slides; original magnification 100×).

On the other hand, in BSCC cases, the agreement between biopsies and surgical specimens was poor to very good for TSR (AC1 statistic 0.6604 [95% CI 0.1752–1.000]) (Figure 1C,D). It was good to very good for large cell nests and tumor budding (AC1 statistic: 0.8759 [95% CI 0.6212–1.000]) for both variables) (Figure 3A–F), whereas it was poor to very good for stroma type (AC1 statistic: 0. 3647 [95% CI −0.2646–0.9940]) (Figure 3A,B). 

### 3.3. Clinical and Histopathological Parameters with a Prognostic Value in Patients with Laryngeal Basaloid and Conventional SCCs

**Figure 4:**
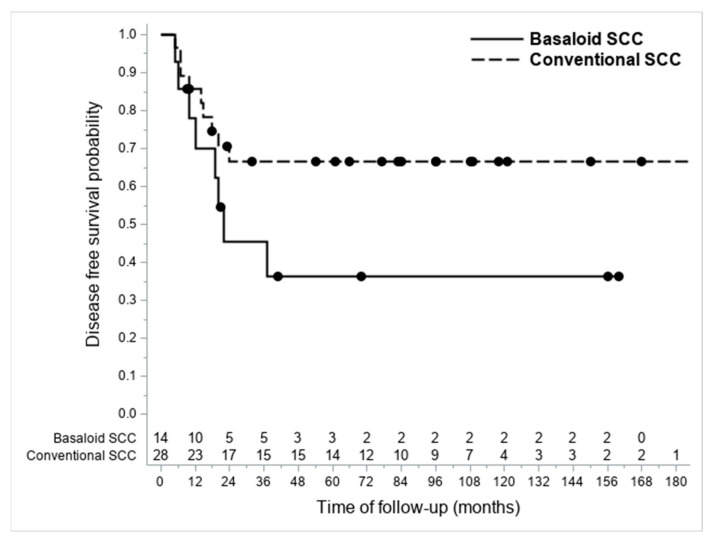
Kaplan-Meier graph showing differences in DFS between considered BSCCs and conventional SCCs.

A higher nodal metastasis percentage was highlighted in patients with BSCCs (57.1% vs. 28.6%; *p* = 0.0723). The recurrence rate resulted as 33.5% and 63.6% in the conventional SCC and BSCC groups, respectively. The DFS probability was higher though not significant in patients with conventional SCCs (see Figure 4) (univariate Cox regression: not adjusted *p* = 0.1229; age-adjusted *p* = 0.7792) (Table 2). 

Table 3 and Table 4 summarize the results of Cox univariate regression model based on DFS for patients with laryngeal basaloid and conventional SCCs, respectively.

**Figure 5:**
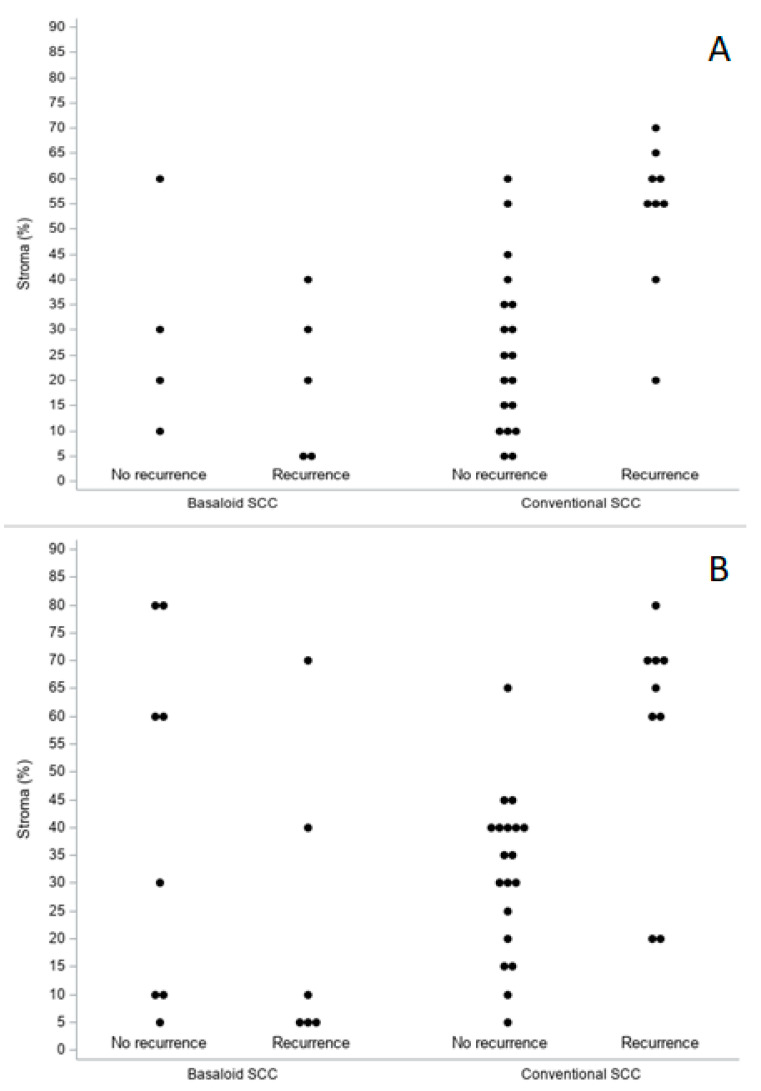
Biopsies (**A**) and surgical specimens (**B**). TSR distribution (percentage of stroma) by histology and prognosis.

Age was the only clinical feature statistically significant in predicting recurrence in the BSCC group (HR per year: 1.16, 95% CI 1.021–1.327; *p* = 0.0235). The other clinical features, as well as the considered histopathological variables (both on biopsies and surgical specimens), did not reach statistical significance. Figure 5 highlights the quantitative values of TSR determined at the level of the biopsies (Figure 5A) and of the definitive histological material (Figure 5B) both in the study group (BSCCs) and in the control one (conventional SCCs), stratified from the point of view of prognosis.

On the other hand, in conventional SCC cases, in addition to age, which retained its negative prognostic value (HR per year: 1.154, 95% CI 1.036–1.286; *p* = 0.0096), TSR also emerged as being a predictor of recurrence. TSR low/stroma-rich cases, when evaluated on biopsies, were associated with a higher probability of recurrence (HR: 10.463, 95% CI 2.156–50.785; *p* = 0.0036). Accordingly, the same feature, as evaluated on surgical specimens, emerged as being associated with a shorter DFS (HR: 10.131, 95% CI 2.082–49.308; *p* = 0.0041). In conventional SCC surgical specimens, TSR retained its statistical prognostic significance in our multivariate model (HR: 5.890, 95% CI 1.099–31.555; *p* = 0.0384) (Table 4). 

## 4. Discussion

Only a limited number of studies used methods able to compare the oncogenic and oncosuppressive biologic mechanisms behind BSCCs and conventional SCCs [16,17,18,19,20,21,22,23,24]. Our group investigated the expression of CD105 [25,26] in head and neck BSCCs and compared it with that in conventional SCCs [19]. The comparison of CD105-associated neoangiogenesis suggested a similar biological behavior between head and neck BSCCs and site- and stage-matched conventional SCCs. TSR and CD105-assessed neoangionesis evaluated in pretreatment biopsies and in matched surgical specimens of conventional laryngeal SCC were previously studied by our group. A mutual relation between TSR and neoangiogenesis emerged, as well as an independent prognostic effect, possibly implying a common biomolecular background [12]. It could be speculated that the activation and differentiation of peritumoral mesenchyma may give rise to stroma changes, including neoangiogenesis and desmoplasia, which appeared to support cancer progression.

Our group firstly investigated angiogenin [27] expression in a series of 12 head and neck BSCCs and in 24 site- and stage-matched conventional SCCs [22]. Endothelial angiogenin expression did not differ significantly in head and neck BSCCs and SCCs; angiogenin expression in carcinoma cells was significantly lower in head and neck BSCCs than in SCCs. Marioni et al. [20] determined immunoreactivity to survivin, a member of the family of inhibitors of apoptosis [28], in nine laryngeal BSCCs and nine site- and stage-matched SCCs. In both primary laryngeal BSCCs and SCCs, and in their nodal metastases, a nuclear subcellular localization of survivin prevailed. No significant difference in mean survivin expression between primary BSCCs and SCCs was found. Nuclear survivin expression was significantly higher in BSCCs associated with disease recurrence and poor prognosis. Deniz et al. [29] analyzed the expression of proliferating cell nuclear antigen (PCNA) and bcl-2 protein in 15 patients with laryngeal BSCC and 15 stage- and site-matched controls with conventional SCC. No significant differences between the two groups in the PCNA index or the frequency of bcl-2 overexpression were proved. Further insights into the molecular biology of laryngeal BSCC were provided by Calli et al. [30] with their retrospective study on the immunohistochemical expression of p63 in a cohort of 22 cases. This research group found p63 expression in most BSCC specimens (72.7%). Moreover, p63 expression was higher in cases without lymph node metastases than in those with nodal involvement, thus supporting an inverse association between p63 expression and lymph node status in BSCC. Similarly, Salerno and colleagues [31] described the oncosuppressive role of p27kip1 in laryngeal BSCC. In their cohort of 16 BSCCs, they found a higher p27kip1 expression in patients with no evidence of recurrent disease at follow-up than in those who died of disease. Moreover, at multivariate analysis, low levels of p27kip1 expression were significantly associated with poor prognosis, also confirming the oncosuppressive role of this protein in laryngeal BSCC. 

To the best of our knowledge, only one previous very preliminary investigation compared tumor-stroma proportions in four cases of laryngeal BSCC vs. 81 conventional SCCs; inferential statistics were not applicable [23]. In the present study, TSR evaluated in preoperative biopsies and matched resection specimens of laryngeal carcinoma showed a poor to very good concordance between the two different tissue samples in the BSCC group and a moderate to very good concordance in the conventional SCC one, confirming previous similar results obtained for the first time in laryngeal cancer by our research team [12]. Furthermore, for conventional SCC cases only, a prognostic importance for this parameter also emerged, as a low TSR in biopsies and operative specimens was significantly associated with a lower DFS (HR: 10.131; *p* = 0.0041). In our multivariate model, considering conventional SCC surgical specimens, TSR retained its statistical prognostic significance. A similar analysis was conducted previously on esophageal carcinoma, in which the TSR score on biopsies was concordant with surgical specimens in 81% of the cases [32]. More recently, in another paper on invasive breast cancer, the correlation coefficients between biopsy-TSR and resection specimens-TSR scores assessed by two pathologists were 0.45 and 0.37, and the sensitivity and specificity of the detection of stroma-rich tumors were 64.1% and 66.7% [33]. If validated in larger series, TSR evaluation on biopsy specimens can provide information on the TSR of the tumor in its entirety, possibly facilitating preoperative risk assessment, estimation of response to non-surgical therapies, and decision-making.

Recent research has underlined many markers that can be assessed using HE-stained sections to improve risk stratification of head and neck SCC [34]. Our group also assessed the concordance in terms of tumor budding, large cell nests, and stroma type between biopsies and surgical specimens in laryngeal basaloid and conventional SCCs. In the conventional SCC group, agreement was moderate to very good for large cell nests and tumor budding and was good to very good for stroma type. In the BSCC series, agreement was good to very good for large cell nests and tumor budding, but conversely was poor to very good for stroma type. In the literature, cell nest size in laryngeal SCC was assessed only by Karpathiou et al. [13]: in their series, the presence of large cell nests was significantly associated with response to chemotherapy and marginally associated with stroma-poor tumors. Cell nest size, budding activity, and TSR have previously been described as surrogate parameters for tumor aggressiveness in head and neck SCC [9,10,11,12,13]. These parameters are all related to a crosstalk between cancer cells and associated stroma, representing an epiphenomenon of loss of cancer cell adhesion and also of epithelial-mesenchymal transition, a biological process that enables tumor cells to acquire a more infiltrative phenotype [13,14]. The suboptimal result in terms of concordance when dealing with the stroma type in BSCCs could be explained by the fact that this subtype is intrinsically a stroma-poor tumor, with an expansive growth, an absence of budding, and a fibrous (hyalinized) stroma. Moreover, the peculiar morphological features of this histotype may represent a potential bias in evaluating TSR, stroma type, the presence of large neoplastic nests, and tumor budding: BSCC is composed, according to the WHO definition [35], o rounded nests with smooth borders, closely apposed with thin lines of hyalinized stroma between them. These characteristics could partially explain the concordance between biopsy and resection specimens in the evaluation of stromal-related histopathological variables.

The analysis of clinical outcomes in this study showed a higher nodal metastasis presence (57.1% vs. 32.1%) and a lower DFS in BSCCs compared to conventional SCCs (Figure 4); these differences between the two subtypes resulted not significant at the univariate analysis, probably owing to the limited number of the BSCC series. Interestingly, in our study, the only factor that could affect prognosis within the BSCC group was age. In addition, this was reasonable because of the limited number of cases, but it might suggest that, within a histologically high-risk category, prognosis may be more likely to depend on the patient’s clinical status rather than on the histological features investigated herein. Of note, age also resulted as a risk factor for recurrence within the conventional SCC group and was the only demographic parameter significantly different in the BSCC group, compared to the conventional SCCs (mean: 70.9 vs. 64.9 years; *p* = 0.0405) in our stage matched analysis.

The main weaknesses of our study concern the retrospective setting and limited number of cases considered. The main strengths lie in the homogeneity of the series of patients considered as (I) two well-defined histotypes (BSCC and conventional SCC) located in a single head and neck structure (the larynx) were considered; (II) BSCC and SCC groups were accurately matched using a pathological staging criterion; (III) all patients were treated consecutively with primary laryngeal surgery by the same team in the same institution; (IV) both preoperative biopsies and surgical samples were assessed in most BSCC and SCC patients; (V) several non-conventional pathological variables were evaluated (TSR, stroma type, large cell nests and tumor budding, and TILs) by head and neck dedicated pathologists; (VI) oncological follow-up criteria were standardized. Another strong point of the study is the decision to also consider laryngeal biopsies. Since during biopsy only a small portion of a solid tumor is sampled, it is not necessarily representative of the entire neoplasm. The very limited size of the biopsy material of laryngeal malignancies is sometimes a critical aspect in pathological diagnosis [36]. In particular, there is a paucity of data on the prognostic significance of biopsies in laryngeal BSCC. To the best of our knowledge, this study was the first to examine the prognostic roles of TSR in paired biopsies and surgical specimens of laryngeal BSCC vs. conventional SCC.

## 5. Conclusions

This exploratory investigation found that laryngeal BSCC showed a lower DFS than stage-matched conventional SCC, even if statistical significance was not reached. TSR showed a good concordance between the evaluation of preoperative biopsies and matched resection specimens of laryngeal carcinoma, in particular in the conventional SCC group. TSR, evaluated in pretreatment laryngeal biopsies and in the entire excised tumor, displayed a prognostic effect in terms of DFS in conventional LSCC cases but not in BSCC ones. Age was the only variable driving the prognosis in the laryngeal BSCC group. Further studies on large multi-centric series are mandatory to confirm these findings and to overcome the limited sample size of laryngeal BSCC due to its rarity.

## Figures and Tables

**Table 1:** Descriptive statistics of demographic and clinical-pathological variables, and their comparison between laryngeal BSCC and conventional SCC.

	Group		
	Basaloid SCC (*n* = 14)	Conventional SCC (*n* = 28)	*p* Value
**Age, years**			
Mean (SD)	70.9 (8.5)	64.9 (8.6)	**0.0405**
Median (Range)	71.0 (51.0–84.0)	65.5 (49.0–80.0)	
**pT**			
T1–2	10 (71.4)	14 (50.0)	0.1859
T1	3	4	
T2	7	10	
T3–4	4 (28.6)	14 (50.0)	
T3	1	8	
T4	3	6	
**Nodal status**			
N0	6 (42.9)	20 (71.4)	0.0723
N+	8 (57.1)	8 (28.6)	
**Stage**			
I–II	6 (42.9)	12 (42.9)	1.0000
III–IV	8 (57.1)	16 (57.1)	
**TSR (biopsy)**			
TSR high/stroma poor	8 (88.9)	19 (67.9)	0.3932
TSR low/stroma rich	1 (11.1)	9 (32.1)	
**Stroma type (biopsy)**			
Fibroblastic	5 (55.6)	4 (14.3)	**0.0231**
Fibrotic	4 (44.4)	24 (85.7)	
**Large cell nests (biopsy)**			
Absent	1 (11.1)	5 (17.9)	1.0000
Present	8 (88.9)	23 (82.1)	
**Intratumoral bud count (biopsy)**			
Mean (SD)	0.3 (1.0)	1.5 (2.8)	0.0875
Median (Range)	0.0 (0.0–3.0)	0.0 (0.0–13.0)	
**Intratumoral budding (biopsy)**			
Low-risk	9 (100.0)	25 (089.3)	0.5622
High-risk	0 (0.0)	3 (10.7)	
**TSR (surgical specimen)**			
TSR high/stroma poor	9 (64.3)	19 (67.9)	1.0000
TSR low/stroma rich	5 (35.7)	9 (32.1%)	
**Stroma type (surgical specimen)**			
Fibroblastic	3 (21.4)	3 (10.7)	0.3825
Fibrotic	11 (78.6)	25 (89.3)	
**Large cell nests (surgical specimen)**			
Absent	0 (0.0)	8 (28.6)	**0.0368**
Present	14 (100.0)	20 (71.4)	
**Peritumoral bud count (surgical specimen)**			
Mean (SD)	1.3 (2.3)	2.9 (4.0)	0.1031
Median (Range)	0.0 (0.0–8.0)	2.0 (0.0–18.0)	
**Peritumoral budding (surgical specimen)**			
Low-risk	13 (92.9)	23 (82.1)	0.6448
High-risk	1 (7.1)	5 (17.9)	
**TILs % (surgical specimen)**			
Mean (SD)	22.1 (16.5)	33.4 (20.3)	0.1131
Median (Range)	27.5 (0.0–40.0)	30.0 (10.0–80.0)	
**Pattern of invasion (surgical specimen)**			
Expansive	7 (50.0)	20 (71.4)	0.1719
Infiltrative	7 (50.0)	8 (28.6)	
**Adjuvant radiation therapy**			
No	7 (50.0)	16 (57.1)	0.6611
Yes	7 (50.0)	12 (42.9)	

Five missing biopsies in BSCC group. Student’s *t* test (age) or Mann–Whitney test for quantitative variables, chi-square (pT, nodal status, stage, pattern of invasion surgical specimen, adjuvant radiation therapy), or Fisher’s test for categorical variables; SCC: squamous cell carcinoma; SD: standard deviation.

**Table 2:** Univariate Cox’s regression model based on the histotype.

	Outcome		Not Adjusted		Age-Adjusted	
	NED (*n* = 25)	REC (*n* = 17)	*p*-Value	HR (95% CI)	*p*-Value	HR (95% CI)
**Group**						
Conventional SCC	19 (76.0)	9 (52.9)		1		
BSCC	6 (24.0)	8 (47.1)	0.1229	2.120 (0.816; 5.509)	0.7992	1.141 (0.412; 3.159)

CI: confidence interval; HR: hazard ratio; NED: no evidence of disease; REC: recurrence.

**Table 3:** Descriptive statistics and univariate Cox’s regression results on demographic and clinical-pathological variables within the BSCC group.

	Outcome			
	NED (*n* = 6)	REC (*n* = 8)	*p*-Value	HR (95% CI)
**Age, years**				
Mean (SD)	67.0 (9.5)	73.8 (6.9)	0.0235	1.164 (1.021; 1.327)
Median (Range)	67.5 (51.0–79.0)	74.5 (64.0–84.0)		
**pT**				
pT1–2	4 (66.7)	6 (75.0)	0.9746	1
pT3–4	2 (33.3)	2 (25.0)		0.974 (0.194; 4.879)
**Nodal status**				
N0	1 (16.7)	5 (62.5)	0.3248	1
N+	5 (83.3)	3 (37.5)		0.486 (0.116; 2.042)
**Stage**				
I–II	1 (16.7)	5 (62.5)	0.3248	1
III–IV	5 (83.3)	3 (37.5)		0.486 (0.11; 2.042)
**TSR (biopsy)**				
TSR high/stroma poor	5 (100.0)	3 (75.0)	0.1688	1
TSR low/stroma rich	0 (000.0)	1 (25.0)		23.958 (0.260; 2209.900)
**Stroma type (biopsy)**				
Fibroblastic	2 (40.0)	3 (75.0)	0.7371	1
Fibrotic	3 (60.0)	1 (25.0)		0.676 (0.069; 6.657)
**Large cell nests (biopsy)**				
Absent	1 (20.0)	0 (0.0)	0.9366	1
Present	4 (80.0)	4 (100.0)		1.150 (0.037; 35.736)
**Intratumoral bud count (biopsy)**				
Mean (SD)	0.0 (0.0)	0.8 (1.5)	0.1722	1.906 (0.755; 4.814)
Median (Range)	0.0 (0.0–0.0)	0.0 (0.0–3.0)		
**Intratumoral budding (biopsy)**				
Low-risk	5 (100.0)	4 (100.0)		-
**TSR (surgical specimen)**				
TSR high/stroma poor	5 (83.3)	4 (50.0)	0.1977	1
TSR low/stroma rich	1 (16.7)	4 (50.0)		2.523 (0.617; 10.312)
**Stroma type (surgical specimen)**				
Fibroblastic	2 (33.3)	1 (12.5)	0.3178	1
Fibrotic	4 (66.7)	7 (87.5)		2.923 (0.356; 23.982)
**Large cell nests (surgical specimen)**				
Present	6 (100.0)	8 (100.0)		-
**Peritumoral bud count (surgical specimen)**				
Mean (SD)	2.2 (3.1)	0.6 (1.4)	0.3146	0.782 (0.484; 1.263)
Median (Range)	1.0 (0.0–8.0)	0.0 (0.0–4.0)		
**Peritumoral budding (surgical specimen)**				
Low-risk	5 (83.3)	8 (100.0)	0.5878	1
High-risk	1 (16.7)	0 (000.0)		0.429 (0.020; 9.155)
**TILs % (surgical specimen)**				
Mean (SD)	30.0 (9.5)	16.3 (18.7)	0.4772	0.983 (0.937; 1.031)
Median (Range)	30.0 (15.0–40.0)	7.5 (0.0–40.0)		
**Pattern of invasion (surgical specimen)**				
Expansive	2 (33.3)	5 (62.5)	0.1518	1
Infiltrative	4 (66.7)	3 (37.5)		0.341 (0.078; 1.486)
**Adjuvant radiation therapy**				
No	4 (66.7)	3 (37.5)	0.5086	1
Yes	2 (33.3)	5 (62.5)		1.625 (0.385; 6.863)

CI: confidence interval; HR: hazard ratio; NED: no evidence of disease; REC: recurrence; SD: standard deviation. One missing biopsy in NED group and 4 in REC one; Firth’s correction applied for TSR and large cell nests in biopsy and peritumoral budding in surgical specimen.

**Table 4:** Descriptive statistics: univariate and multivariate Cox’s regression results on demographic and clinical-pathological variables within the conventional SCC group.

	Outcome		Univariate		Multivariate	
	NED (*n* = 19)	REC (*n* = 9)	*p*-Value	HR (95% CI)	*p*-Value	HR (95% CI)
**Age, years**						
Mean (SD)	62.3 (8.3)	70.6 (6.5)	0.0096	1.154 (1.036; 1.286)	0.0961	1.099 (0.983; 1.229)
Median (Range)	63.0 (49.0–78.0)	69.0 (61.0–80.0)				
**pT**						
pT1–2	11 (57.9)	3 (33.3)	0.2391	1		
pT3–4	8 (42.1)	6 (66.7)		2.301 (0.575; 9.219)		
**Nodal status**						
N0	15 (78.9)	5 (55.6)	0.1509	1		
N+	4 (21.1)	4 (44.4)		2.633 (0.703; 9.866)		
**Stage**						
I–II	9 (47.4)	3 (33.3)	0.5001	1		
III–IV	10 (52.6)	6 (66.7)		1.611 (0.403; 6.448)		
**TSR (biopsy)**						
TSR high/stroma poor	17 (89.5)	2 (22.2)	0.0036	1		
TSR low/stroma rich	2 (10.5)	7 (77.8)		10.463 (2.156; 50.785)		
**Stroma type (biopsy)**						
Fibroblastic	2 (10.5)	2 (22.2)	0.4622	1		
Fibrotic	17 (89.5)	7 (77.8)		0.554 (0.115; 2.674)		
**Large cell nests (biopsy)**						
Absent	3 (15.8)	2 (22.2)	0.8397	1		
Present	16 (84.2)	7 (77.8)		0.850 (0.175; 4.116)		
**Intratumoral bud count (biopsy)**						
Mean (SD)	1.6 (3.2)	1.3 (1.7)	0.7421	0.956 (0.733; 1.248)		
Median (Range)	0.0 (0.0–13.0)	1.0 (0.0–5.0)				
**Intratumoral budding (biopsy)**						
Low-risk	17 (89.5)	8 (88.9)	0.9330	1		
High-risk	2 (10.5)	1 (11.1)		0.915 (0.114; 7.324)		
**TSR (surgical specimen)**						
TSR high/stroma poor	17 (89.5)	2 (22.2)	0.0041	1	0.0384	1
TSR low/stroma rich	2 (10.5)	7 (77.8)		10.131 (2.082; 49.308)		5.890 (1.099; 31.555)
**Stroma type (surgical specimen)**						
Fibroblastic	2 (10.5)	1 (11.1)	0.9330	1		
Fibrotic	17 (89.5)	8 (88.9)		1.093 (0.137; 8.754)		
**Large cell nests (surgical specimen)**						
Absent	5 (26.3)	3 (33.3)	0.8247	1		
Present	14 (73.7)	6 (66.7)		0.855 (0.213; 3.431)		
**Peritumoral bud count (surgical specimen)**						
Mean (SD)	1.8 (2.5)	5.1 (5.7)	0.0546	1.124 (0.998; 1.266)		
Median (Range)	1.0 (0.0–10.0)	3.0 (0.0–18.0)				
**Peritumoral budding (surgical specimen)**						
Low-risk	17 (89.5)	6 (66.7)	0.2673	1		
High-risk	2 (10.5)	3 (33.3)		2.193 (0.548; 8.783)		
**TILs % (surgical specimen)**						
Mean (SD)	37.6 (21.0)	24.4 (16.1)	0.0957	0.966 (0.928; 1.006)		
Median (Range)	40.0 (10.0–80.0)	20.0 (10.0–60.0)				
**Pattern of invasion (surgical specimen)**						
Expansive	15 (78.9)	5 (55.6)	0.3191	1		
Infiltrative	4 (21.1)	4 (44.4)		1.952 (0.524; 7.276)		
**Adjuvant radiation therapy**						
No	12 (63.2)	4 (44.4)	0.2978	1		
Yes	7 (36.8)	5 (55.6)		2.013 (0.539; 7.515)		

CI: confidence interval; HR: hazard ratio; NED: no evidence of disease; REC: recurrence; SD: standard deviation.

## Data Availability

The datasets generated and analyzed during the current study are available on reasonable request.
